# Molecular Characterization of an Endozoicomonas-Like Organism Causing Infection in the King Scallop (Pecten maximus L.)

**DOI:** 10.1128/AEM.00952-17

**Published:** 2018-01-17

**Authors:** Irene Cano, Ronny van Aerle, Stuart Ross, David W. Verner-Jeffreys, Richard K. Paley, Georgina S. E. Rimmer, David Ryder, Patrick Hooper, David Stone, Stephen W. Feist

**Affiliations:** a Cefas Weymouth Laboratory, Centre for Environment, Fisheries and Aquaculture Science, Weymouth, Dorset, United Kingdom; INRS—Institut Armand-Frappier

**Keywords:** Endozoicomonas, marine protected area, king scallop, metagenomics, Rickettsia-like organisms

## Abstract

One of the fastest growing fisheries in the UK is the king scallop (Pecten maximus L.), also currently rated as the second most valuable fishery. Mass mortality events in scallops have been reported worldwide, often with the causative agent(s) remaining uncharacterized. In May 2013 and 2014, two mass mortality events affecting king scallops were recorded in the Lyme Bay marine protected area (MPA) in Southwest England. Histopathological examination showed gill epithelial tissues infected with intracellular microcolonies (IMCs) of bacteria resembling Rickettsia-like organisms (RLOs), often with bacteria released in vascular spaces. Large colonies were associated with cellular and tissue disruption of the gills. Ultrastructural examination confirmed the intracellular location of these organisms in affected epithelial cells. The 16S rRNA gene sequences of the putative IMCs obtained from infected king scallop gill samples, collected from both mortality events, were identical and had a 99.4% identity to 16S rRNA gene sequences obtained from “Candidatus Endonucleobacter bathymodioli” and 95% with Endozoicomonas species. *In situ* hybridization assays using 16S rRNA gene probes confirmed the presence of the sequenced IMC gene in the gill tissues. Additional DNA sequences of the bacterium were obtained using high-throughput (Illumina) sequencing, and bioinformatic analysis identified over 1,000 genes with high similarity to protein sequences from Endozoicomonas spp. (ranging from 77 to 87% identity). Specific PCR assays were developed and applied to screen for the presence of IMC 16S rRNA gene sequences in king scallop gill tissues collected at the Lyme Bay MPA during 2015 and 2016. There was 100% prevalence of the IMCs in these gill tissues, and the 16S rRNA gene sequences identified were identical to the sequence found during the previous mortality event.

**IMPORTANCE** Molluscan mass mortalities associated with IMCs have been reported worldwide for many years; however, apart from histological and ultrastructural characterization, characterization of the etiological agents is limited. In the present work, we provide detailed molecular characterization of an Endozoicomonas-like organism (ELO) associated with an important commercial scallop species.

## INTRODUCTION

The king scallop (Pecten maximus L.) is a valuable seafood product with large established markets within Europe and the wider world. In the UK, the majority of king scallops produced for the table market are fished from natural stocks (1). UK vessels generated £58.3 million in 2014 from landings of just 38,500 tonnes, and this fishery is increasing more rapidly than any other commercially targeted shellfish species (2). Marine protected areas (MPAs) have been established to protect threatened species and important habitats through the restriction of human activities. In the case of Lyme Bay, off the South coast of England, this has been shown to be beneficial for the recuperation of scallop stocks within the MPA (3).

However, two mass mortality events of the king scallop, within the Lyme Bay MPA, were recorded by commercial fishermen in June 2013 and May 2014 (Fish Health Inspectorate, Cefas, personal communication). Histological examinations showed a severe infection of intracellular microcolonies (IMCs) of bacteria as well as diffuse infections affecting the gill tissue of specimens collected during both mass mortality events. King scallop mass mortalities associated with IMC infections have been reported occasionally for many years in fisheries around the English Channel (S. Feist, unpublished data). In 1988, mass mortalities of scallops were reported from North Brittany in France (4), and subsequent surveys showed a 100% prevalence of intracellular bacteria in scallops at that location based on histological examination (5).

IMCs associated with pathogenicity and mortalities have been reported from mollusc species worldwide. These include the Pacific oyster (Magallana gigas, previously Crassostrea gigas), common orient clam (Meretrix lusoria), Ezo giant scallop (Patinopecten yessoensis), and abalone (Haliotis rufescens) (6, 7). However, most IMC infections in marine molluscs are asymptomatic (8).

Despite the likely economic impact of IMCs in highly valuable mollusc species, these bacteria remain largely uncharacterized. IMCs were previously known as Rickettsia-like organisms (RLO). RLOs are a group of phenotypically similar organisms. The general phenotypic characteristics of the organisms previously designated RLOs in marine bivalve molluscs were: obligate parasites of the host cell, polymorphic in shape, between 0.4 and 2 μm, bacteria-like in their propagative modes (i.e., binary fission and budding), and able to form intracytoplasmic inclusions (9). For consistency, prokaryotes with these, or a majority of these, features in bivalve molluscs were commonly designated RLOs. However, molecular studies have increasingly shown that the use of the term “RLO” can be misleading. Many of the intracellular bacterial infections of molluscs and other animals are not caused by true members of the order Rickettsiales and include Gram-negative bacteria from both the Alphaproteobacteria and Gammaproteobacteria subclasses of Proteobacteria (10). Recently, the rapid expansion in bacterial genome data has provided insights into the adaptive, diversifying, and reductive evolutionary processes that occur during host adaptation (11).

Very little is known about IMC infectivity in molluscs. There have been some attempts to purify and characterize IMCs associated with molluscs, including their likely antigenic properties (12, 13). Experimental reproduction of symptoms has been reported for an IMC infecting Tapes japonica and transmission of withering syndrome to abalones (14, 15). Furthermore, the transmission routes and host range are poorly understood. Chlamydia-like and IMC bacteria have been observed in king scallop larvae by transmission electron microscopy (TEM) (16), and epidemiological investigation suggests horizontal transmission for the dissemination of RLO in scallops (17).

Our understanding of the mollusc-infecting IMC is limited by a lack of published molecular data from these organisms, as well as the identification of virulence markers. In the present study, we characterized the IMC associated with scallop mass mortalities, showing it was a member of the Gammaproteobacteria subgroup of the Proteobacteria. Using a specific PCR diagnostic tool as part of an epidemiological survey, we also demonstrated its high prevalence in the scallop stock in the Lyme Bay MPA.

## RESULTS

### Histopathology.

**FIG 1:**
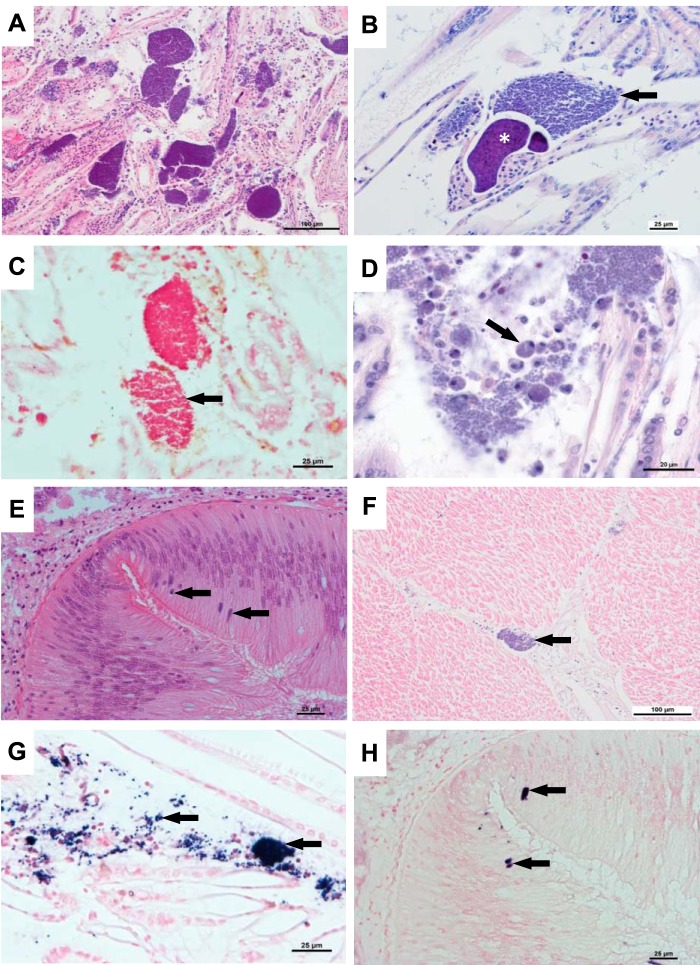
ELO infection in P. maximus. (A) Histological section showing a region of severe disruption of the gill associated with the presence of numerous accumulations and disrupted microcolonies of bacteria (IMC). Hematoxylin and eosin (H&E) stain. (B) Detail of gill section showing type I (arrow) and type II (asterisk) colonies. H&E stain. (C) Gram-negative ELO colonies. Gram stain. (D) Section showing diffuse infiltration of hemocytes (arrow) associated with ELO infection. H&E stain. (E) ELO microcolonies (arrows) in the intestinal epithelium. H&E stain. (F) Section of the adductor muscle showing focal ELO infection (arrow) in the intermuscular connective tissue. H&E stain. (G) *In situ* hybridization (ISH) of the ELO 16S rRNA gene in the gill. Labeling is observed as dark-blue staining (arrows). (H) Similar section to that depicted in panel E. ISH labeling of ELO microcolonies in the same location (arrows). Bars = 100 μm (A and F), 25 μm (B, C, E, G, and H), and 20 μm (D).

Scallops sampled in the Lyme Bay MPA during 2013 and 2014 showed an infection of branchial tissue with intracellular microcolonies of a range of sizes (colony areas ranging from 1 × 10^3^ to 3 × 10^5^ μm^2^) (Fig. 1A), often with bacteria present in vascular spaces. Large colonies were associated with cellular and tissue disruption of the gills. Following a previously published scoring system to semiquantify the intensity of RLO infection in P. maximus (4), and where severe infection represents an average number of 40 colonies counted on a section consisting of 4 transversal branchial pieces of equal surface, the intensity of infections in the current study ranged from moderate to severe.

Two types of bacterial microcolony morphology previously described in king scallops (4) were observed in the infected gills. Type I colonies were composed of tightly packed globular cells, and type II colonies characteristically contained intensely basophilic small rods (Fig. 1B). Both types of colonies were composed of Gram-negative bacteria (Fig. 1C).

Diffuse invasion of the gill epithelia by hemocytes was observed only in extreme cases of severe IMC growth associated with the disruption of the gill tissue (Fig. 1A and D).

In addition to the gill infections, IMCs were also observed in the intestinal epithelium and in the abductor muscle in a single specimen (Fig. 1E and F). From samples taken in 2013, one animal showed low levels of a coccidian infection in the adductor muscle, and a further two animals showed infection with a different coccidian in the kidney at low intensity. From samples taken in 2014, there was no evidence of any toxic effects in the tissues examined. Mild renal coccidiosis was seen in two animals. Chlamydia-like organisms (CLOs) were observed in low numbers in the digestive gland of three animals. Eight animals showed focal hemocyte accumulation in various tissues.

### *In situ* hybridization of scallop IMCs.

Strong *in situ* hybridization (ISH) labeling was detected in the IMCs in the gill epithelium (Fig. 1G), including in both type I and II bacterial microcolonies. ISH-positive cells were also observed at low prevalence in the intestinal epithelium (Fig. 1H). No labeling was observed in negative controls of infected scallop tissue in the absence of a specific probe.

### Ultrastructural examinations.

**FIG 2:**
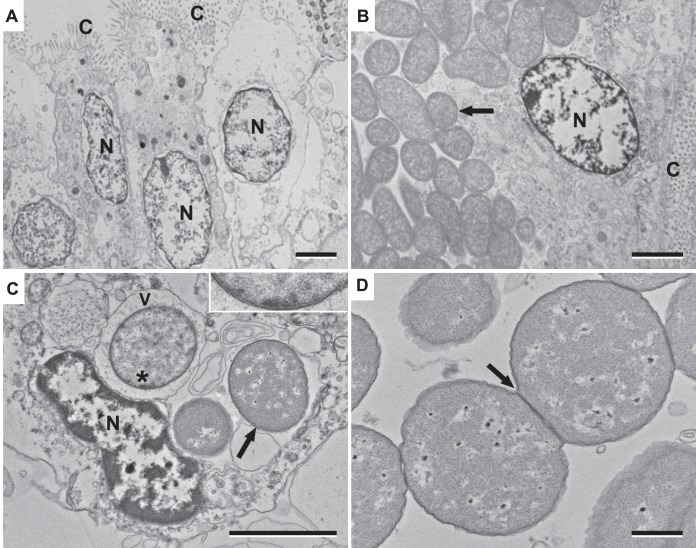
Ultrastructural examination of ELO in P. maximus. (A) Transmission electron micrograph (TEM) image of uninfected branchial epithelial cell. N, cell nucleus; C, cilia. (B) TEM of infected branchial epithelial cell showing cytoplasmic ELOs (arrow). (C) TEM of a host hemocyte containing ELOs (arrow) within cytoplasmic vacuoles. V, vacuole. Top right inset, detail of bacterial inner and outer cell membranes. (D) ELO propagation by binary fission (arrow). Bars = 2 μm (A to C) and 500 nm (D).

Using TEM, the bacteria were observed to be dispersed throughout the cytoplasm of gill epithelial cells (Fig. 2B) or within vacuoles in the cytoplasm of host cells, occasionally in hemocytes (Fig. 2C). In heavy infections, large numbers of bacteria appear to be released following cell rupture. The bacteria showed an inner and outer membrane (Fig. 2C, inset), and bacterial propagation by binary fission was observed (Fig. 2D).

### Lyme Bay MPA survey.

Severely infected gills, seen in eight animals from site A sampled in 2015, showed macroscopic subtle focal or multifocal lesions, which appeared to correspond to the presence of particularly large bacterial colonies seen histologically. Copepods were observed in 14 out of 30 samples collected from site B, with no associated macroscopic pathology.

Histological examination showed IMC infection in gills in 96% and 100% of the samples taken from sites A and B, respectively. Severity was related to the number and size of the IMC inclusions. Severe IMC lesions were observed in 24.1% and 6.6% of the animals sampled from sites A and B, respectively, and the rest showed mild lesions. Both IMC type I and II microcolonies were observed in the histological examination. Cardiac hemocytosis was observed at high prevalence (65.5% and 53.3% from each site, respectively). Other parasites were detected histologically at very low numbers and prevalence.

IMCs were seen in 100% of the scallops sampled in 2016 in gill sections. Furthermore, based on histological examination and ISH of 45 individuals, IMCs were observed in low numbers in the intestinal epithelium of 1 individual (Fig. 1E and H) and in the adductor muscle of 3 individuals (Fig. 1F). Other parasites, including trichodinids, gregarines, and parasitic worms, were also observed (Table 1).

**TABLE 1:** Lyme Bay MPA scallop survey, summary of the gross examination, histology, and ELO detection by PCR

Characteristic	No. by sampling yr		
	2015		2016
	Site A (*n* = 29)	Site B (*n* = 30)	Site A (*n* = 45)
Gross pathology			
Pale gills	3	0	0
IMC	8	0	0
Copepod	0	14	0
Histology			
IMC^*a*^			
Mild	13	23	
Moderate	8	5	10
Severe	7	2	35
Cardiac hemocytosis			
Mild	16	5	
Moderate		5	
Severe	3	6	
Digenea parasite	1		
Coccidian	1		
Trichodina	1		2
Gill granulomatosis		1	
Gregarine			1
Parasitic worm			2
ELO PCR positive	29	30	45

a IMC, intracellular microcolonies of bacteria.

**FIG 3:**
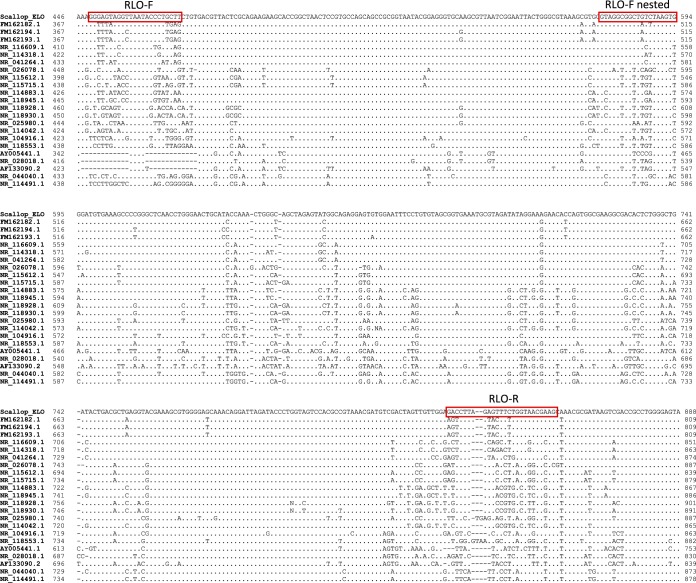
Multiple-sequence alignment of the region of the 16S rRNA gene for which specific king scallop Endozoicomonas-like organism (ELO) primers were developed. NCBI GenBank accession numbers: “Candidatus Endonucleobacter bathymodioli,” FM162182.1, FM162194.1, and FM162193.1; Endozoicomonas montiporae, NR_116609.1; Endozoicomonas numazuensis, NR_114318.1; Endozoicomonas elysicola, NR_041264.1; Pseudomonas aeruginosa, NR_026078.1; Pseudomonas syringae, NR_115612.1; Pseudomonas fluorescens, NR_115715.1; Aeromonas hydrophila, NR_114883.1; Aeromonas salmonicida, NR_118945.1; Vibrio parahaemolyticus, NR_118928.1; Vibrio vulnificus, NR_118930.1; Piscirickettsia salmonis, NR_025980.1; Escherichia coli, NR_114042.1; Coxiella burnetii, NR_104916.1; Wolbachia persica, NR_118553.1; Ehrlichia risticii, AY005441.1; Rickettsia rickettsii, NR_028018.1; agent of withering syndrome, AF133090.2; Ralstonia solanacearum, NR_044040.1; and Burkholderia cepacia, NR_114491.1. Dots represent nucleotides identical to those of the scallop ELO; −, missing nucleotides. The ELO-F, ELO-R, and ELO-F nested primers are highlighted in the red boxes.

The diagnostic PCR described below in Materials and Methods (Fig. 3) was used to amplify the 16S rRNA gene of the IMC affecting king scallops from gill tissues. A band of the expected size was amplified in all the samples analyzed, from both sites and years, after the first round of PCR. Sequencing of the PCR product showed a single organism that was 100% identical to the samples sequenced in 2013 and 2014.

### The king scallop IMC is an Endozoicomonas-like organism.

**FIG 4:**
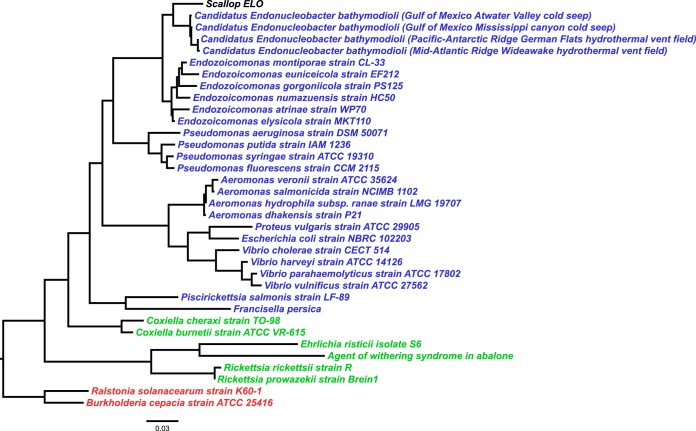
Maximum likelihood tree showing phylogenetic relationships among the king scallop ELO and a selection of alphaproteobacteria, betaproteobacteria, and gammaproteobacteria (highlighted in green, red, and blue, respectively) based on 16S rRNA gene sequences. NCBI GenBank accession numbers: Endozoicomonas montiporae, NR_116609.1; Endozoicomonas numazuensis, NR_114318.1; Endozoicomonas euniceicola, NR_109684.2; Endozoicomonas gorgoniicola, NR_109685.1; Endozoicomonas atrinae, NR_134024.1; Endozoicomonas elysicola, NR_041264.1; “Candidatus Endonucleobacter bathymodioli,” FM162182.1, FM244838.1, FM162193.1, and FM162194.1; Pseudomonas aeruginosa, NR_026078.1; Pseudomonas syringae, NR_115612.1; Pseudomonas fluorescens, NR_115715.1; Pseudomonas putida, NR_043424.1; Aeromonas dhakensis, NR_042155.1; Aeromonas veronii, NR_119045.1; Aeromonas hydrophila subsp. *ranae*, NR_114883.1; Aeromonas salmonicida, NR_118945.1; Vibrio parahaemolyticus, NR_118928.1; Vibrio vulnificus, NR_118930.1; Vibrio cholerae, NR_044853.1; Vibrio harveyi, NR_119054.1; Proteus vulgaris, NR_115878.1; Piscirickettsia salmonis, NR_025980.1; Escherichia coli, NR_114042.1; Coxiella cheraxi, NR_116014.1; Coxiella burnetii, NR_104916.1; Wolbachia persica, NR_118553.1; Ehrlichia risticii, AY005441.1; Rickettsia rickettsii, NR_028018.1; Rickettsia prowazekii, NR_044656.1; agent of withering syndrome, AF133090.2; Ralstonia solanacearum, NR_044040.1; and Burkholderia cepacia, NR_114491.1.

A consensus fragment of 1,408 bp of the IMC 16S rRNA gene was obtained from gill homogenates of king scallops sampled in 2013 by PCR amplification and sequencing. This sequence showed 95% similarity with the 16S rRNA gene sequence of Endozoicomonas elysicola strain MKT110 (NCBI accession no. NR_041264.1) and 99.4% with “Candidatus Endonucleobacter bathymodioli” (NCBI accession no. FM162182.1). Amplified 16S rRNA gene sequences obtained from samples taken during the 2014 mortality event showed 100% similarity to those from 2013, based on a consensus fragment of 600 bp of the scallop IMC 16S rRNA gene. Phylogenetic analysis based on the 16S rRNA gene and 6 bacterial housekeeping genes demonstrated that the king scallop IMC is a member of the Gammaproteobacteria subgroup of the Proteobacteria (Fig. 4). The universal chlamydial PCR was negative for all the samples analyzed.

### Metagenomics.

A total of 2,569,653 raw Illumina reads were obtained, and after quality trimming, 2,203,598 paired and 349,797 unpaired reads remained (Table S1 in the supplemental material). *De novo* assembly of the metagenome resulted in 80,333 contigs, with a total of 48 Mbp of assembled sequence, ranging from 200 to 56,063 bp in length (mean length, 599 bp; *N*_50_, 549 bp).

Annotation of assembled contigs resulted in the identification of 28,760 genes, or protein-coding regions, of which 21% (6,051 protein-coding regions) were matched against proteins in NCBI's collection of nonredundant proteins (i.e., the nr database). Of those sequences which matched known species, around 70% appeared to represent the host, given they showed high similarity to sequences from another bivalve mollusc species (Crassostrea gigas). The remaining 1,926 sequences (31.8% of the annotated sequences) showed high similarity to species in the Endozoicomonas genus and were therefore thought to represent sequences from the IMC organism. The full set of results is published online (https://doi.org/10.14466/CefasDataHub.36).

The putative IMC nucleotide sequences accounted for 3.27 Mbp of sequence, with a median contig length of 17.5 kb and a median coverage of 5×. Only those contigs which had more than 3× coverage and which were longer than 1 kb were analyzed. This approach led to the majority of the IMC sequences being retained (3.24 Mbp of assembled sequence), while accounting for 4.83 Mbp of sequence in total. Within this smaller subset of longer higher-coverage sequences, there were 3,790 genes, of which 1,676 showed high similarity to those of Endozoicomonas species and 46 appeared to represent the host, given they matched sequences from another bivalve mollusc species (Crassostrea gigas).

Bacterial rRNA gene analysis of the putative IMC genome using METAXA2 identified only one 16S rRNA gene sequence (1,462 bp), which was identical to the 1,408 bp of the IMC 16S rRNA gene sequence obtained by PCR amplification described earlier. The phylogenetic relationships of this sequence to a number of closely related bacteria are shown in Fig. 4. This 16S rRNA gene fragment showed 93.3% sequence similarity with Endozoicomonas numazuensis strain HC50 16S rRNA (NCBI accession no. NR_114318.1). Additionally, one 23S rRNA gene sequence (2,896 bp) was identified that showed 90.4% sequence similarity with the 23S rRNA gene of Endozoicomonas montiporae CL-33 (NCBI accession no. CP013251.1).

**FIG 5:**
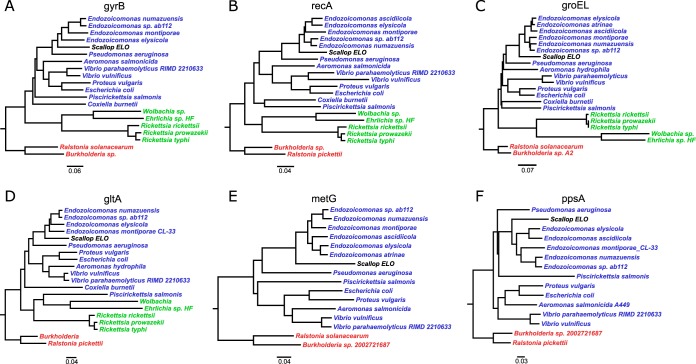
Neighbor-joining tree showing phylogenetic relationships among the king scallop ELO and a selection of alphaproteobacteria, betaproteobacteria, and gammaproteobacteria (highlighted in green, red, and blue, respectively) based on six housekeeping gene sequences, including those for DNA gyrase subunit B (*gyrB*) (A), recombinase A (*recA*) (B), 60-kDa chaperonin (*groEL*) (C), citrate synthase (*gltA*) (D), methionine-tRNA ligase (*metG*) (E), and phosphoenolpyruvate synthase (*ppsA*) (F). See Table 2 for more details on the selected bacterial species and the corresponding proteins.

Consistently, other (non-rRNA gene) scallop IMC gene sequences were most similar to those of Endozoicomonas species, ranging from 77 to 87% identity, followed by Pseudomonas spp. (61 to 80%) and Aeromonas spp. (58 to 76%) (Fig. 5). Similarities to Rickettsiales sequences, placed in the Alphaproteobacteria subgroup of the Proteobacteria, ranged between 50 and 61%.

### Preliminary putative functional genomics of the scallop ELO.

A total of 3.24 Mbp of the 3.27-Mbp-long metagenomics-constructed scallop Endozoicomonas-like organism (ELO) genome showed high sequence similarity to sequences from various Endozoicomonas species. Out of 3,711 proteins identified in the assembly, 1,438 proteins were associated with a biological process in the Gene Ontology database. A table with the protein accession numbers and the associated records in the InterPro and Gene Ontology databases is provided in the Data Set S1 in the supplemental material. Sequences related to genes involved in nitrate and nitrogen metabolism, synthesis of amino acids, aerobic respiration, carbohydrate metabolic processes, oxidation-reduction processes, and determinants of virulence, such as type III secretion needle-protein-like and hemolysin BL-binding component genes, among others, were all highly similar to those of members of the genus Endozoicomonas.

## DISCUSSION

This study characterizes an Endozoicomonas-like species infecting the gill of king scallops. Partial 16S rRNA gene sequence analysis placed the scallop ELO in the Gammaproteobacteria subgroup of the Proteobacteria. This was confirmed by high-throughput sequencing of infected gill samples and metagenomic analysis. Phylogenetic analysis, based on the 16S rRNA gene, showed a high similarity of the scallop ELO with other intracellular bacteria infecting bivalves: the “Candidatus Endonucleobacter bathymodioli,” which invades the nuclei of deep-sea bathymodiolin mussels from hydrothermal vents and cold seeps (18), and the intranuclear pathogen nuclear inclusion x (NIX), infecting nonciliated branchial epithelia associated with massive mortalities of the Pacific razor clam (Siliqua patula) (19, 20).

In this study, we compare a metagenome-constructed scallop ELO genome with published genomes of other bacteria. A large number of genes were similar to those present in species of the symbiotic genus Endozoicomonas. The genus Endozoicomonas was first described in 2007 (21). Endozoicomonas species typically reside in aggregates within host tissues, have a free-living stage, show signs of host and local adaptation, participate in host-associated protein and carbohydrate transport and cycling, and harbor a high degree of genomic plasticity (22).

The complete genomes of three Endozoicomonas type strains from E. elysicola, E. montiporae, and E. numazuensis have been sequenced and assembled. Endozoicomonas species genomes are relatively large, ranging from 5.6 Mb for E. montiporae to 6.83 Mb for E. atrinae (23). In this study, the scallop metagenome-constructed ELO genome resulted in a draft genome sequence of 4.83 Mb, of which 3.24 Mbp of assembled sequence showed highly significant protein-level similarity with Endozoicomonas species. The draft scallop ELO genome is thus smaller than other sequenced Endozoicomonas species. Endozoicomonas bacteria are emerging as extremely diverse and flexible symbionts of numerous marine hosts, such as sponges, corals, molluscs (24), and fish (22). It remains unclear how symbiotic Endozoicomonas species interact with their host (23). True members of the order Rickettsiales share an intimate association with eukaryotic cells, and the relationship with their host is obligate intracellular parasitism or mutualism (10). The loss of regulatory genes causes an increase of virulence in Rickettsia species infecting ticks and mammals (25). In contrast, Endozoicomonas genomes are large and are not streamlined for an obligate endosymbiotic lifestyle, implying that they have free-living stages. However, different Endozoicomonas genotypes might play disparate roles and have diversified in concert with their hosts (26). For example, “Candidatus Endozoicomonas cretensis” is an epitheliocystis-causing pathogen in sharp snout seabream (Diplodus puntazzo) (Walbaum, 1792) responsible for fish larva mortalities (27). Its genome shows loss-of-function genes and insertion sequence expansion, often indicative of adaptation to a new niche or restriction to an alternative lifestyle (27). It is possible that a genetic variant displaying a loss of regulatory genes could play a role in the mass mortalities of king scallops; however, further deep sequencing would be required to confirm the final full genome size of the scallop ELO. The lack of molecular data of intracellular bacteria infecting other bivalves hampers further comparative characterization, including the identification of virulence markers.

Recurrent mass mortality events in the stocks of king scallops around the English Channel have been reported since the 1980s (4). Surveys carried out in Northern Brittany (France) in the 1990s (5) and recently in Southwest England (present study) have shown 100% prevalence of scallop ELO type organisms in the samples analyzed. However, severe pathology, observed as a general disruption of gill epithelial tissue, associated with the ELO growth and infiltrative-type inflammatory responses, characterized as focal or diffuse invasion of injured tissue by immunocytes (28), was only observed in specimens showing severe ELO infection.

It is reasonable to hypothesize that the ELOs could persist at low levels in scallop populations as a symbiont, with replication of the bacterium increasing as a consequence of increased water temperatures during summer months and resulting in pathology and mortality. It is likely, however, that other factors could play a role in influencing pathogenicity in the host (e.g., algal toxins during bloom events), but these have yet to be identified.

Sporadically, IMCs were observed in tissues other than gills, including the intestinal epithelium and the adductor muscle, although in low numbers and with limited host response. By means of ISH, using an ELO-specific probe, we have shown that the IMC in the intestinal epithelium is likely to be the same organism as that infecting the gills. Indeed, the sensitivity afforded by using ISH for detecting low numbers of the target organism is likely to show that these infections, although predominant in the gill, are present throughout the animal.

The establishment of MPAs is designed to protect endangered species and habitats. In the case of Lyme Bay, there is evidence that scallop stocks have increased, providing a sustainable fishery resource for this species, which is now being exploited by diving rather than dredging. Because of the recently detected high prevalence of the ELO infection in scallop stocks in the Lyme Bay MPA, and in order to understand the transmission and pathogenesis of the infection, periodic health assessments would be required, as has been previously suggested (29).

In the present study, we have designed a specific PCR for the detection of the ELOs infecting scallops, which can be used to undertake epidemiological and host range studies. Due to the lack of sequence data for IMCs infecting other bivalves from populations in other locations around the coast, we recommend the use of universal primers (11, 30) together with the specific set of primers designed in this study.

Despite IMCs of bacteria being recognized as significant bivalve pathogens, they remain uncultured in standard media for intracellular organisms or within molluscan cell lines. This has hampered studies on their biology and the ability to demonstrate Koch's postulates and pathogenesis of infection in molluscan hosts. Future studies should address these aspects and relate field observations, potentially including the detection of scallop ELO DNA in the environment, to disease outbreaks.

## MATERIALS AND METHODS

### King scallop mass mortality events in 2013 and 2014 sample collections.

In June 2013, king scallop mortalities were reported from the Lyme Bay MPA. Ten king scallops, measuring approximately 11 cm in shell diameter, were sampled for histology and molecular analysis.

In May 2014, approximately 40% mortality was reported by fishermen operating in the MPA. Thirty king scallops were collected by a commercial fisherman at coordinates 50.65775°N, 2.85776°W (WGS84). Blooms of Pseudo-nitzschia and possibly Phaeocystis were noted at the time of the sampling.

Specimens were observed for gross pathology, and tissues were sampled for histopathology, analysis by molecular biology (PCR amplification and sequencing), and transmission electron microscopy (TEM), as described below.

### Lyme Bay MPA survey in 2015 and 2016.

King scallops were sampled in August 2015 from two sites within the MPA: site A, 50.66668°N, 2.8155°W; and site B, 50.66683°N, 2.86228°W. Thirty and 29 scallops from sites A and B, respectively, were collected by hand by a local fisherman. In 2016, 45 animals were sampled from site A only. Animals were examined for gross pathology and samples taken for subsequent histology and PCR analysis.

### Histopathology.

King scallops were fixed for 24 h in Davidson's seawater fixative, which was prepared from the commercial product PRC/R/205 Davidson sea water fixative stock solution, as per the manufacturer's instruction (PRC Chemicals). Tissues were embedded in paraffin wax using a vacuum infiltration processor, according to standard protocols. Embedded blocks were sectioned at 3- to 4-μm thickness using a rotary microtome, and sections were stained with hematoxylin and eosin (H&E). Sections were examined using a Nikon E800 light microscope and images captured using the Nikon NIS-Elements BR software.

### Electron microscopy.

Gill tissues from IMC-infected king scallops were fixed in 2.5% glutaraldehyde in 0.1 M sodium cacodylate buffer (pH 7.4) for 2 h at room temperature and then rinsed in 0.1 M sodium cacodylate buffer (pH 7.4). Tissues were postfixed for 1 h in 1% osmium tetroxide in 0.1 M sodium cacodylate buffer. Gill tissues were washed in three changes of 0.1 M sodium cacodylate buffer before dehydration through a graded acetone series. Samples were embedded in Agar 100 epoxy (Agar 100 premix kit medium; Agar Scientific) and polymerized overnight at 60°C in an oven. Semithin (1- to 2-μm) sections were stained with toluidine blue for viewing with a light microscope to identify suitable target areas. Ultrathin sections (70 to 90 nm) of these areas were mounted on uncoated copper grids and stained with 2% aqueous uranyl acetate and Reynolds' lead citrate (31). Grids were examined using a JEOL JEM 1400 transmission electron microscope and digital images captured using an AMT XR80 camera and AMTv602 software.

### IMC 16S rRNA gene sequencing.

Ethanol-fixed gill tissues, collected in 2013 and 2014, were homogenized 1:10 in G2 buffer (Qiagen, UK) and digested with 6 milli-Anson units of proteinase K at 55°C for 3 h. DNA was then extracted from 200 μl of homogenized tissue using an EZ1 DNA tissue kit in an EZ1 extraction robot (Qiagen). DNA was eluted in 50 μl of elution buffer, according to the manufacturer's instructions.

A fragment of the scallop IMC 16S rRNA gene was amplified by PCR using the primers FD1 (AGAGTTTGATCCTGGCTCAG) and rP2 (ACGGCTACCTTGTTACGACTT) (11). In parallel, a set of universal primers designed for the detection of Chlamydiales (32) were also tested by PCR. PCRs were performed in a 50-μl reaction volume consisting of 1× GoTaq Flexi buffer (Promega, UK), 2.5 mM MgCl_2_, 1 mM dinucleoside triphosphate (dNTP) mix, 50 pmol forward and reverse primers, 1.25 units of GoTaq DNA polymerase (Promega), and 2.5 μl of the purified DNA. The reaction mixture was overlaid with mineral oil, and, after an initial denaturing step (5 min at 95°C), it was subjected to 35 temperature cycles (1 min at 95°C, 1 min at 55°C, and 1 min at 72°C) in a PTC-225 Peltier thermal cycler (MJ Research, Canada), followed by a final extension step of 10 min at 72°C. PCR products were visualized on 2% agarose gels stained with ethidium bromide.

PCR products were purified using GeneClean (Anachem, UK). Both DNA strands were sequenced using the ABI Prism Dye Terminator cycle sequencing kit (PerkinElmer, UK) on an ABI 310 genetic analyzer. Sequence similarity searches were conducted using blastn (33) and the NCBI nucleotide database.

### Primer design for a king scallop IMC diagnostic PCR.

Specific primers for amplifying a fragment of the scallop IMC 16S rRNA gene were identified using variable regions within the gene based on the multiple-sequence alignment described above (Fig. 3). Primer sequences were selected based on unique specificity to the scallop IMC sequence (predicted melting temperature [*T_m_*], ≥60°C).

PCR primers IMC-F (5′-GGGAGTAGGTTAATACCCTGCTT-3′) and IMC-R (5′-CTTCGTTACCAGAAACTCTAAGGTC-3′) were used for a first round of PCR, followed by a seminested PCR using IMC-F nested (GTAGGCGGCTGTCTAAGTG) and IMC-R, resulting in amplified fragments of 407 bp and 282 bp, respectively. PCRs were performed as described above.

### ISH.

Sequential sections, mounted on silane-treated slides (Sigma-Aldrich, UK), were used for ISH localization of bacterial 16S rRNA gene sequences. ISH assays were carried out as previously described (34). Briefly, deparaffinized and rehydrated sections were permeabilized with proteinase K (100 μg · ml^−1^ in diethyl pyrocarbonate [DEPC]-H_2_O; Promega, UK) for 30 min at 37°C. Hybridization was performed using 600 bp of the scallop IMC 16S rRNA digoxigenin (DIG). The 16S rRNA sequence was labeled with digoxigenin (DIG) by PCR (Roche, UK) using the forward primer S-D-Bact-0008-a-S-20 (5′-AGAGTTTGATCCTGGCTCAG-3′) and reverse primer S-*-Univ-0536-a-A-18 (5′-GWATTACCGCGGCKGCTG-3′) (30). The DIG-labeled probe was denatured at 95°C for 5 min prior to hybridization overnight at 42°C. Posthybridization washes were performed twice with [1times] saline sodium citrate buffer (SSC) with 6 M urea and 0.2% (wt/vol) bovine serum albumin (BSA) for 15 min at 42°C. Tissue sections were blocked with 6% (wt/vol) skimmed milk (Sigma) in Tris-borate (TB) buffer and incubated with an anti-DIG monoclonal antibody conjugated to alkaline phosphatase (Roche) for 1 h. The hybridization signal was detected using nitroblue tetrazolium–5-bromo-4-chloro-3-indolylphosphate (NBT/BCIP; Roche). Nuclei were counterstained with Nuclear Fast Red (Sigma). Tissue sections were dehydrated with 94% ethanol for 30 s, followed by 100% ethanol for 30 s, and then air dried and mounted with a coverslip and mounting medium (Entellan; Merck).

### High-throughput sequencing.

DNA was extracted from two IMC-infected scallop gill samples collected in 2013, as described above, and subjected to high-throughput sequencing using an Illumina MiSeq (2 × 300-bp paired-end protocol) at The Food and Environment Research Agency (FERA). The paired-end sequence reads generated were quality checked using FastQC version 0.11.4 (35) and subsequently trimmed to remove adapter sequences and low-quality bases using Trimmomatic version 0.33 (36). The paired and unpaired reads of both samples were assembled using the SPAdes *de novo* assembly algorithm (37). Prokka (38) was used to identify protein-coding regions in the assembled contigs, which were subsequently aligned against those from other species using Diamond (39) (blastp algorithm; E value < 1e-05) and the full NCBI protein sequence database (nr, version 20160821). In parallel, METAXA2 (40) was used to search for the presence of rRNA sequences in the assembled contig data set.

### Characterization of sequences.

To avoid analyzing partial gene sequences and sequences of organisms with very low representation within the sample, contigs with less than 3× coverage and/or contigs which were shorter than 1 kb were removed prior to further analysis. The remaining 4.83 Mbp of assembled sequences which exceeded these criteria was submitted to the NCBI.

InterProScan version 5.25.64 was used to identify protein domains and match any associated accession numbers in the InterPro database (41). A table mapping the InterPro accession numbers to Gene Ontology terms was downloaded from the Gene Ontology website (42). The Gene Ontology database was then retrieved from the Gene Ontology website, in OWL RDF/XML format, and converted to an SQLite database using the OwlReady2 python module (43). Finally, the InterPro results, the table mapping InterPro results to Gene Ontology terms, and the Gene Ontology database were combined into the same SQLite database, and an SQL query was run to find any protein domains which had been associated with a biological process, as defined in the Gene Ontology database.

### Phylogenetic analysis.

A selection of alphaproteobacteria (including Coxiella burnetii, Ehrlichia risticii, Rickettsia rickettsii, and an agent of withering syndrome), betaproteobacteria (including Ralstonia solanacearum and Burkholderia cepacia), and gammaproteobacteria (including Endozoicomonas spp., “Candidatus Endonucleobacter bathymodioli” spp., Pseudomonas spp., Aeromonas spp., Vibrio spp., Piscirickettsia salmonis, Francisella persica, and Escherichia coli) sequences were downloaded from the NCBI nucleotide database. The sequences were then aligned against the full scallop IMC 16S rRNA sequence using the ClustalW algorithm (44) in MEGA version 7 (45).

Furthermore, a selection of bacterial housekeeping genes, including those for DNA gyrase subunit B (*gyrB*), recombinase A (*recA*), molecular chaperone/chaperonin (*groEL*), citrate (Si)-synthase (*gltA*), methionine-tRNA ligase (*metG*), and phosphoenolpyruvate synthase (*ppsA*) genes, were identified and selected for multiple-species alignments to a selection of representative alphaproteobacteria, betaproteobacteria, and gammaproteobacteria (see Table 2 for a complete list of species and corresponding NCBI GenBank accession numbers), using the ClustalW algorithm (44) in MEGA version 7 (45). Phylogenetic relationships between the scallop IMC and other bacterial sequences were inferred using the maximum likelihood method in MEGA7, and trees were drawn and annotated using FigTree (version 1.4.3; http://tree.bio.ed.ac.uk/software/figtree/).

**TABLE 2:** List of housekeeping genes and their corresponding NCBI GenBank accession numbers for the scallop ELO and a selection of Alphaproteobacteria, Betaproteobacteria, and Gammaproteobacteria used to construct the phylogenetic trees of Fig. 4^*a*^

Organism	Accession no. by housekeeping gene					
	*gyrB*	*recA*	*groEL*	*gltA*	*metG*	*ppsA*
Scallop ELO	PJE79691	PJE80279	PJE78732	PJE80590	PJE80385	PJE79160
E. elysicola	WP_020582222.1	WP_020584428.1	WP_020581046.1	WP_020583787.1	WP_020580433.1	KEI71730.1
E. numazuensis	WP_034840273.1	WP_034834656.1	WP_034832823.1	WP_034837138.1	WP_034833472.1	KEQ12216.1
E. montiporae	WP_034878976.1	WP_034873081.1	WP_034874990.1	AMO55799.1	WP_034874706.1	AMO56485.1
Endozoicomonas sp. ab112	WP_062260709.1	WP_062264698.1	WP_062268132.1	WP_062266761.1	WP_062265063.1	WP_062270171.1
Endozoicomonas ascidiicola		WP_067521539.1	WP_067514952.1		WP_067520035.1	WP_067584669.1
E. atrinae			WP_066014043.1		WP_066018113.1	
A. salmonicida	WP_059112343.1	WP_059114185.1			WP_059167808.1	ABO90573.1
A. hydrophila			WP_065392449.1	OCY04558.1		
V. parahaemolyticus	NP_796393.1	NP_798929.1	WP_069540105.1	NP_797221.1	NP_798448.2	BAC61715.1
V. vulnificus	WP_053543016.1	WP_045592534.1	WP_061057819.1	WP_038939417.1	WP_061058381.1	OJI52689.1
P. vulgaris	WP_036933417.1	WP_036932587.1	WP_036934353.1	WP_036938444.1	WP_036938249.1	KGA60261.1
E. coli	WP_063121202.1	WP_071666031.1	WP_071686894.1	WP_069906759.1	WP_020238642.1	OAC44216.1
Wolbachia	WP_052264704.1	WP_012673345.1	WP_065094753.1	WP_010081982.1	WP_012481739.1	
R. prowazekii	WP_004597896.1	WP_004596976.1	WP_004596265.1	WP_014411765.1	WP_004596363.1	
Rickettsia typhi	WP_011191017.1	WP_011191180.1	WP_011191061.1	WP_014419995.1	WP_011191115.1	
R. rickettsii	WP_014363134.1	WP_014362415.1	WP_014362581.1	WP_012151414.1	WP_014363212.1	
Ehrlichia sp. strain HF	WP_044194822.1	WP_044193970.1	WP_044194315.1	WP_044195590.1	WP_044194094.1	
C. burnetii	WP_043880795.1	WP_005772330.1	WP_040947985.1	WP_032075108.1	WP_012569713.1	
P. aeruginosa	WP_061199402.1	WP_061200254.1	WP_071580146.1	KFL13041.1	WP_058129887.1	KFL10045.1
P. salmonis	WP_017378470.1	WP_017376458.1	WP_016209875.1	WP_017378335.1	WP_027242971.1	ALB21516.1
R. solanacearum	WP_064050680.1		WP_064050138.1		WP_064048779.1	
Burkholderia	WP_039364634.1	WP_059520572.1	WP_069621205.1	WP_010089184.1		AJY41073.1
Ralstonia pickettii		WP_024976563.1		KFL18994.1		KFL23738.1

a *gyrB*, DNA gyrase subunit B; *recA*, recombinase A; *groEL*, molecular chaperone/chaperonin; *gltA*, citrate (Si)-synthase; *metG*, methionine-tRNA ligase; *ppsA*, phosphoenolpyruvate synthase.

### Accession number(s).

The sequence of the scallop IMC 16S rRNA gene has been deposited in NCBI GenBank with accession no. KX780138. The raw sequencing files were deposited in the NCBI Sequence Read Archive (SRA) under BioProject no. PRJNA386592. This whole-genome shotgun project has been deposited at DDBJ/ENA/GenBank under the accession no. NSIT00000000. The version described in this paper is version NSIT01000000. The full annotation table of the contigs can also be retrieved from https://doi.org/10.14466/CefasDataHub.36.

## Supplementary Material

Supplemental material
