# Supplementary material for: Molecular Characterization of an Endozoicomonas-Like Organism Causing Infection in the King Scallop (Pecten maximus L.)
Source: Appl Environ Microbiol. 2018 Jan 17;84(3):e00952-17. doi: 10.1128/AEM.00952-17 (PMC5772249; doi:10.1128/AEM.00952-17)
Supplement: Supplemental material [file supp_84_3_e00952-17__index.html]

Supplemental material 

# Molecular Characterization of an Endozoicomonas-Like Organism Causing Infection in the King Scallop (Pecten maximus L.)

## Supplemental material

- Supplemental file 1 -

  Number of Illumina sequence reads before and after quality trimming (Table S1).

  PDF, 265K
- Supplemental file 2 -

  Analysis of the scallop ELO genome in relation to biological processes (Data Set S1).

  XLSX, 167K
