# Supplementary material for: Molecular Characterization of an Endozoicomonas-Like Organism Causing Infection in the King Scallop (Pecten maximus L.)
Source: Appl Environ Microbiol. 2018 Jan 17;84(3):e00952-17. doi: 10.1128/AEM.00952-17 (PMC5772249; doi:10.1128/AEM.00952-17)
Supplement: Supplemental material [file AEM.00952-17_zam003188289s1.pdf]

TABLE S1 Number of Illumina sequence reads before and after quality-trimming.

Sample	Raw paired reads	Trimmed paired reads	Orphan reads (R1)	Orphan read (R2)
2C	1,352,636	1,168,855	173,635	1,395
3C	1,217,017	1,034,743	173,533	1,234
